# Miscellaneous

**Published:** 1890-05

**Authors:** 


### MISCELLANEOUS.

### PROMOTION OF HEALTH.

Dr. Bell, in his work on climatology, says that mere climate has little influence on health, and thus sums up the subject: "After all that has been stated of the effects of atmosphere in high altitudes, or at the level of the sea, the influence of forests and ocean, of sea coasts and interior places, humidity and dryness, cold and heat, the winds, electricity, and ozone, and whatever other conditions, the paramount considerations for promotion of health are an abundance of pure air and sunshine and outdoor exercise. Without these no climate is promotive of health propitious for cure of disease; and with them, it is safe to say, the human powers of accommodation are such that it is difficult to distinguish the peculiarities of any climate by their joint result on the health and longevity of its subjects."

### THE ANALYSIS OF A CIGARETTE.

*Good Health* has made an analysis of a cigarette. "The tobacco was found to be strongly impregnated with opium, while the wrapper, which was warranted to be rice paper, was proved to be the most ordinary quality of paper whitened with arsenic. The two poisons combined were present in sufficient quantities to create in the smoker a habit of using opium without his being aware of it, his craving for which can only be satisfied by an incessant consumption of cigarettes." The State should prohibit the making and vending of poisonous cigarettes, as well as poisonous alcoholic beverages, and for kindred reasons.

WHERE CHOCOLATE COMES FROM.—Chocolate is made from the seeds of the Theobroma cocoa tree which is found only in tropical climates, and bears a fruit somewhat like a cucumber in shape, inside of which are the brownish seeds or beans, which form the cocoa beans of commerce. The principal constituent of these beans is a soft, solid oil, called cocoa butter, and their attractive principle is theobromine, analogous to the caffeine in coffee. There is but very little pure chocolate in the market, owing to the great medicinal value of the cocoa butter or oil which is expressed in the grinding, and cheaper, less nutritious oil supplied. One of the best ways to buy cocoa or chocolate, it is said, is to purchase what are called "cocoa nibs," which are the beans crushed in fragments, but not ground, for the ground chocolate is frequently adulterated with roasted hazel nuts or almonds, ricemeal, oatmeal and other ingredients.

The best chocolate is prepared by first burying the fruit until the pulp is decayed and only the beans are left. The beans are roasted and the shells removed. The chocolate is then ground between stones, the friction heat of the grinding melting it so that it is a soft molten mass as it drips from the stones and is poured into moulds. The melted chocolate is pressed in cloth until all the oil is expelled; the sediment is ground very slowly to prevent remelting it, and the powder bolted like flour through silken sieves, and then it is called cocoa which makes a lighter, less nourishing, but more easily digested beverage than chocolate.

### AN ELEPHANT'S CUNNING.

The following story is told by Dr. Romanes :—"An elephant was chained to a tree in the compound. The driver made an oven at a short distance, in which he put his rice cakes to bake ; then covered them with stones and grass, and went away. When he was gone, the elephant with his trunk unfastened the chain round his foot, went to the oven and uncovered it, took out and ate the cakes, re-covered the oven with the stones and grass as before, and went back to his place. He could not fasten the chain round his own foot, so he twisted it round and round it, in order to look the same, and when the driver returned the elephant was standing with his back to the oven. The driver went to his cakes, discovered the theft, and looking round, caught the elephant's eye as he looked back over his shoulder out of the corner of it. Instantly he detected the culprit, and condign punishment followed. The whole occurrence was witnessed from the windows by the family."

---

Longevity of Animals.—The average age of cats is 25 years ; of squirrels and hares, 7 or 8 years ; rabbits, 7 ; a bear rarely exceeds 20 years ; a dog lives 20 years ; a wolf, 20 ; a fox, 14 to 16 ; lions are long lived, the one known by the name of Pompey lived to the age of 70 ; elephants have been known, it is asserted, to live to the great age of 400 years. When Alexander the Great had conquered Porus, king of India, he took a great elephant, which had fought very valiantly for the king and named him Ajax, dedicating him to the sun and let him go with this inscription : "Alexander, the son of Jupiter, dedicated Ajax to the sun." The elephant was found with this inscription 350 years after. Pigs have been known to live to the age of 20, and the rhinoceros to 20 ; a horse has been known to live to the age of 62, but average from 25 to 30 ; camels sometimes live to the age of 100 ; stags are very long lived ; sheep seldom exceed the age of 10 ; cows live about 15 years. Cuvier considered it probable that whales sometimes live 4,000 years ; the dolphin and porpoise attain the age of 40 ; an eagle died at Vienna at the age of 104 ; ravens frequently reach the age of 100 ; swans have been known to live 300 years. Mr. Malerton has the skeleton of a swan that attained the age of 200 years. Pelicans are long lived ; the tortoise has been known to live to 107.

A New Discovery.—In the course of conversation at Cornell University, Mr. Atkinson, the Boston economist, stated that a New England genius has recently discovered a cheap method of dissolving zinc by combining it with hydrogen, and producing a solution called zinc-water. This liquid, if applied to certain woods, notably, white wood, makes it absolutely fire-proof, and at a low cost. Mr. Atkinson regards this discovery as one of the most important of the age, and one that will surely revolutionize fire insurance, as well as immensely decrease the loss by fire. The invention is kept secret for the present. Only one foreigner —Sir Lyon Playfair, the English scientist—knows of it. He corroborates all that is claimed for the invention, and says the inventor is a bungling chemist, but that he has a faculty of blundering into the choicest secrets in nature's laboratory. As soon as patents are perfected and capital interested, zinc-water will become an article of commerce.

### A PRACTICAL JOKE.

Most squirrels keep two or more stores of food. Wood, the British naturalist, tells of a friend who found one of these reserve stores which a squirrel had provided for an exigency, and the friend, in a moment of thoughtlessness, determined to play a joke on the squirrel. He accordingly replaced the nuts by small round stones, and carefully concealed all evidences of his visit. One cold day in winter, he passed the spot, and found that the squirrel had called there a short time previously. This he knew by the fact that ten inches of snow had been scratched from the top of the hole, outside of which the stones had been cast by the disappointed animal. This struck the joker with remorse. He said: "*I never felt the folly of practical joking so much in my life.* Fancy the poor little fellow, nipped with cold and scanty food, but foreseeing a long winter, resolved to economize his little hoard as long as possible. Fancy him at last determined to break this—perhaps his last—magazine, and cheerily brushing away the snow, fully confident that a good meal awaited him as the reward of his cold job, and, after all, finding nothing but stones. *I never felt more mean and ashamed in my life*, and really would have given a guinea to have known that injured squirrel's address. He should have had as fine a lot of nuts as would have put him beyond the reach of poverty had he lived to be as old as Methuselah."—*Globe Democrat.*

